# Health-Related Quality of Life and Return to Work after Surgery for Spinal Schwannoma: A Population-Based Cohort Study

**DOI:** 10.3390/cancers16101882

**Published:** 2024-05-15

**Authors:** Aman Singh, Ann-Christin von Vogelsang, Victor Gabriel El-Hajj, Ali Buwaider, Alexander Fletcher-Sandersjöö, Jenny Pettersson-Segerlind, Erik Edström, Adrian Elmi-Terander

**Affiliations:** 1 Department of Clinical Neuroscience, Karolinska Institute, 171 76 Stockholm, Sweden; 2 Department of Surgical Sciences, Uppsala University, 752 36 Uppsala, Sweden; 3 Department of Neurosurgery, Karolinska University Hospital, 171 76 Stockholm, Sweden; 4 Sweden Capio Spine Center Stockholm, Löwenströmska Hospital, 194 89 Upplands-Väsby, Sweden; 5 Department of Medical Sciences, Örebro University, 701 82 Örebro, Sweden

**Keywords:** schwannoma, spine, spinal schwannoma, neurosurgery, patient-reported outcomes, health-related quality of life, return to work

## Abstract

**Simple Summary:**

Spinal schwannomas are the second most common primary intradural spinal tumor. Although these tumors are histologically benign, they can cause spinal cord compression with acute or chronic neurological deficits. Health-related quality of life (HRQoL) can be described as quality of life relative to a person’s health or disease status and HRQoL measures may therefore be considered as measures of self-perceived status. Despite the fact that some studies have evaluated the neurological outcomes after surgery for spinal schwannomas, no studies have been conducted on HRQoL and return to work after surgery. In this population-based cohort study, 94 cases of surgically treated spinal schwannomas were followed for a median 7.3 years [4.8–10.6] to assess their HRQoL compared to a sample of the general population. We found that HRQoL was equal between the spinal schwannoma sample and the general population sample in all but one dimension; men in the spinal schwannoma sample reported more moderate problems in the usual activities dimension than men in the general population. The frequency of return to work was 94%. Thus, surgery of spinal schwannomas should be considered a safe procedure with good long-term HRQoL.

**Abstract:**

Spinal schwannomas are the second most common primary intradural spinal tumor. This study aimed to assess health-related quality of life (HRQoL) and the frequency of return to work after the surgical treatment of spinal schwannomas. HRQoL was compared to a sample of the general population. Patients operated for spinal schwannomas between 2006 and 2020 were identified in a previous study and those alive at follow-up (171 of 180) were asked to participate. Ninety-four (56%) responded and were included in this study. Data were compared to the Stockholm Public Health Survey 2006, a cross-sectional survey of a representative sample of the general population. An analysis for any potential non-response bias was performed and showed no significant differences between the groups. HRQoL was equal between the spinal schwannoma sample and the general population sample in all but one dimension; men in the spinal schwannoma sample reported more moderate problems in the usual activities dimension than men in the general population (*p* = 0.020). In the schwannoma sample, there were no significant differences between men and women in either of the dimensions EQ-5D_index_ or EQ_VAS_. Before surgery, a total of 71 (76%) were working full-time and after surgery almost all (94%) returned to work, most of them within 3 months of surgery. Eighty-nine (95%) of the patients responded that they would accept the surgery for their spinal schwannoma if asked again today. To conclude, surgical treatment of spinal schwannomas is associated with good HRQoL and with a high frequency of return to work.

## 1. Introduction

Schwannomas are WHO grade 1 nerve sheath tumors that may affect peripheral, spinal, or cranial nerves [1,2,3,4]. Spinal schwannomas comprise 25% of all intradural spinal cord tumors [2,5,6] with an incidence of 0.3–0.7 per 100,000 [7]. Although benign, large schwannomas can compress nerve roots or the spinal cord, resulting in neurological deficits. Surgery with the aim of gross total resection is the treatment of choice [2,8,9]. Surgical resection is performed to improve the neurological function or halt neurological deterioration and alleviate pain. Surgical treatment of spinal schwannomas is typically associated with postoperative neurological improvements [1]. However, there are few studies on patient-reported outcomes, health-related quality of life (HRQoL) or return to work after surgery for spinal schwannomas [10,11].

HRQoL can be described as quality of life relative to a person’s health or disease status [12], and HRQoL measures may therefore be considered as measures of self-perceived health status [13]. HRQoL is dynamic, subjective and multidimensional [12]. Most conceptualizations of HRQoL include different dimensions of functioning, such as physical functioning, social functioning, role functioning and mental health and, further, patients’ perceptions of general health and symptoms [14]. In the Swedish general population, HRQoL reporting differs between sexes. Women generally report more problems within the dimensions anxiety/depression and pain/discomfort and have lower overall HRQoL in comparison to men of the same ages. Moreover, the number of reported problems increases with age [15]. Subsequently, when HRQoL comparisons are made with the general population, the samples need to be matched by both sex and age.

Previous studies have also highlighted that an individual’s ability to work and the possibility of returning to work after surgery have a great impact on quality of life [15,16,17,18,19,20]

Given the paucity of studies on the topic, the aim of this study was to explore long-term HRQoL and return to work in a consecutive cohort of patients surgically treated for spinal schwannomas. The same cohort was previously investigated for neurological outcomes after surgery [1]. 

## 2. Materials and Methods

This population-based cohort study compared HRQoL data from a spinal schwannoma sample with a general population sample. The HRQoL data and employment data were self-reported in EQ-5D-3L and a study-specific questionnaire.

### 2.1. Samples

#### 2.1.1. Spinal Schwannoma Sample 

**Figure 1:**
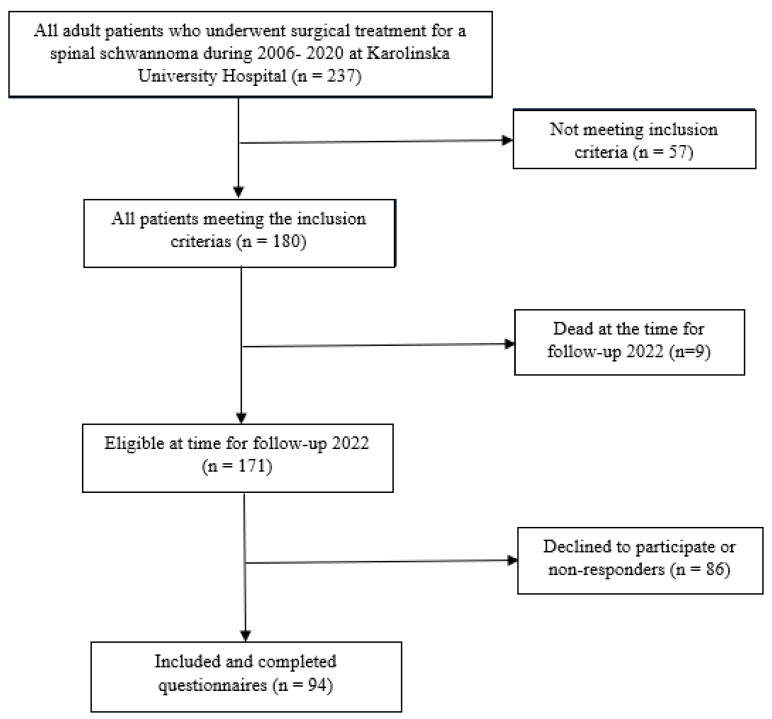
Flowchart of the patient inclusion process.

All adult (≥18 years) patients operated for a spinal schwannoma at the Karolinska University Hospital during a period of 15 years (2006–2020) were identified in a previous study including 180 patients [1]. Of these 180 patients, 171 were still alive in 2022 and were contacted with a request for participation in this follow-up study. A total of 86 patients declined to participate or did not respond. Thus, 94 spinal schwannoma patients were included in the study (55% of eligible patients; Figure 1).

#### 2.1.2. General Population Sample

To compare HRQoL data with the general population, data from the Stockholm Public Health Survey 2006 were used. This was a cross-sectional survey of a representative sample of the general population in Stockholm County. A self-reported postal questionnaire, including the EQ-5D-3L instrument, was sent to 57,000 adult persons aged 18–84 years, with a response rate of 61%. Anonymized raw data were obtained, and for each one of the 94 spinal schwannoma patients that answered the EQ-5D-3L, three control subjects were randomly selected and individually matched by sex and age by the statistical program SPSS (Version 25, IBM, Armonk, NY, USA). Consequently, 282 individuals were included in the general population sample, intended to mirror the population in the Stockholm region. Three women in the spinal schwannoma sample were older than 84 years (aged 85, 87 and 91 years); their controls were therefore matched with the oldest controls, aged 84 years old.

### 2.2. Measures

#### 2.2.1. EQ-5D-3L

The generic HRQoL questionnaire EQ-5D-3L consists of two parts: a descriptive system where the respondents classify their health in five dimensions (mobility, self-care, usual activities, pain/discomfort and anxiety/depression) within three severity levels (no problems, moderate problems or severe problems), and a visual analogue scale (EQ_VAS_) [21]. The response in each dimension of the descriptive system generates a 5-digit value which can be indexed into a single overall HRQoL value, the EQ-5D_index_, where 0 represents dead and 1 represents full health. On EQ_VAS_, the respondents rate their current health between the two anchor points 0 (worst imaginable health) and 100 (best imaginable health). The recall period is the day of completion of the questionnaire. In this study, the United Kingdom (UK) value set was used to calculate the EQ-5D_index_ [22] to enable international comparisons of index values.

#### 2.2.2. Study-Specific Questionnaire 

A study-specific questionnaire was designed with multiple choice questions. The first part of the questionnaire regarded neurological symptoms (motor deficit, sensory deficit, balance, and incontinence) and how these symptoms had changed compared to preoperative status. The patients were also asked whether they would accept to undergo the same surgery if they had been offered it today. Questions were also asked about comorbidities and current medication due to residual symptoms of the tumor. The final part of the questionnaire concerned employment status, sick-leave and return to work after surgery.

#### 2.2.3. Clinical Parameters

Clinical parameters including the modified McCormick score (mMC), symptom duration, tumor recurrence and details of surgical procedure were derived from previously published data [1]. 

### 2.3. Data Analysis

A non-response analysis was conducted, considering all baseline variables (Supplementary Table S1).

EQ-5D-3L data were compared between the spinal schwannoma sample and the general population sample, and sub-group comparisons were made between males and females. To analyze differences between groups, a Fisher’s exact test and an independent samples median test with Yates’s continuity correction were used. Moderate and severe levels on EQ-5D-3L dimensions were collapsed before analysis. Statistical significance was set at *p* < 0.05.

### 2.4. Ethical Considerations

A signed informed consent was obtained from each participant in the spinal schwannoma sample. Anonymized data from the Stockholm Public Health Survey were obtained after ethical approval. The Stockholm Public Health Survey contains data from individuals who have provided consent. The study was approved by the Regional and National Ethical Review Board (Dnr: 2016/1708-31/4 and 2021-05249).

## 3. Results

### 3.1. Baseline and Characteristics of the Sample

In total, 94 patients were included in this study. The median age at the time of surgery was 52 [42–64] years and 45 (48%) were men. At follow-up, the median age was 59 [52–71]. The median follow-up time after surgery was 7.3 years [4.8–11]. The most common presenting symptom was pain, and the median time between symptom debut and surgery was 12 months. Considering neurological function according to the mMC scale, 40 patients (42%) improved, 44 (47%) were unchanged and 10 (11%) worsened. Notably, 35 patients (37%) were mMC I before surgery and could therefore not improve. The most common laminectomy ranges were two (59%) and three levels (23%). A laminoplasty was performed in 51 (54%) cases (Table 1).

### 3.2. Comparison of HRQoL between the Spinal Schwannoma Sample and the General Population Sample

HRQoL was equal between the spinal schwannoma sample and the general population sample in all but one dimension; men in the spinal schwannoma sample reported significantly more problems in the usual activities dimension than the men in the general population sample (*p* = 0.020) (Table 2). In this study, women in both samples reported more problems than the men in the pain/discomfort and anxiety/depression dimensions, but the differences were only significant in the general population sample (*p* = 0.043 and *p* = 0,050, respectively). In the schwannoma sample, there were no significant differences between men and women in any dimension of EQ-5D or EQ_VAS_ (Table 2).

### 3.3. Employment Status and Return to Work after Spinal Schwannoma Surgery

**Figure 2:**
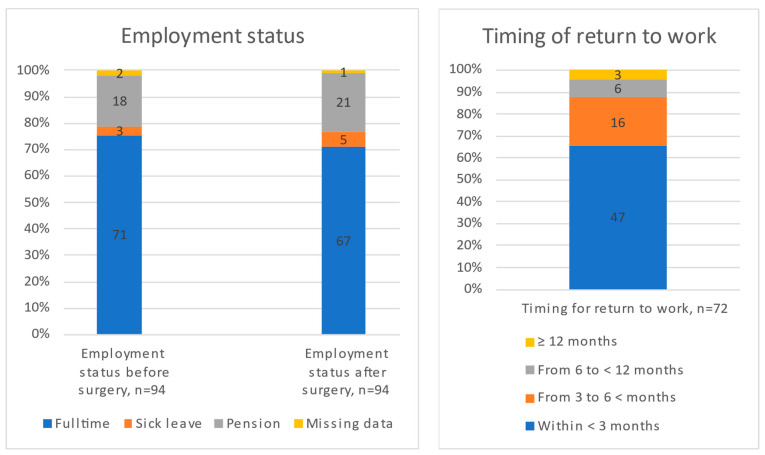
Employment status before and after surgery and timing of return to work.

Before surgery, a total of 71 (76%) of the spinal schwannoma patients were working full-time (Figure 2). Of those who worked before surgery, 67 (94%) had returned to work at follow-up, while 3 patients (4%) had received the old age pension. The majority returned to work within 3 months (70%) (Figure 2). At follow-up, 5 patients were on sick leave, but the cause was unknown.

### 3.4. Remaining Symptoms and Patient Reported Outcomes 

Sixty-four (68%) patients reported at least one remaining symptom in the study-specific questionnaire (Table 3). Thirty-eight (40%) reported improvements in their symptoms postoperatively, thirty-five (37%) reported no change in their symptoms and seventeen (18%) patients felt their symptoms had worsened postoperatively. Four (4.3%) patients did not answer this question. Eighty-nine (95%) of the patients responded that they would accept the surgery if asked again today. Five patients would not accept the surgery if it was offered today. Three of those five said no because of a complication, while two said no because of the absence of improvement or a worsened neurological status.

### 3.5. Comorbidity

The Charlson comorbidity index was in median 1 [0–2], indicating that the schwannoma patients had good general health (Table 1).

### 3.6. Medication

Sixty (64%) patients reported that they did not use any medication related to the surgery (Table 3). Thirty (32%) patients used non-prescription pain medication such as NSAID or Paracetamol after surgery. None of the patients used morphine derivates or spasticity (Baclofen) medication. Three (3.2%) patients used medication for neuropathic pain (Gabapentin or Pregabalin).

## 4. Discussion

In this study, the HRQoL of patients surgically treated for spinal schwannoma at long-term follow-up did not differ from the general population. Following surgical treatment for spinal schwannomas, patients reported pain, anxiety, mobility, and self-care scores comparable to those of the general population based on EQ5D assessments. A majority of the patients working before surgery returned to work (94%), and most of them within 3 months (70%). The most used medications were non-prescription analgesics (32%), and only 3.2% used prescription analgesics, such as neuropathic pain medication. None of the patients used morphine derivates or any spasticity medication.

Only a few studies have addressed the HRQoL after surgery for spinal schwannomas; most of them are studies in mixed groups of different intradural spinal tumors [23]. In a study of benign intradural extramedullary tumors, patients experienced statistically significant improvements regarding pain, work, mood, general activity, and enjoyment of life [24]. Viereck et al., reported that a resection of intradural spinal tumors improved HRQoL by decreasing patient disability and pain and improving each of the EQ-5D dimensions [11]. These findings are in accordance with our results. One important aspect of schwannomas, in contrast to meningiomas and myxopapillary ependymomas, is that schwannomas are often associated with radicular pain [8,9,25] and that the nerve root cannot always be preserved [1]. Thus, good surgical outcomes cannot be taken for granted, especially regarding long-term sensory deficits. The schwannoma sample reported similar general health problems to the matched controls. This may be a general pattern following surgery for a curable pathology. Previous studies have shown that non-curable conditions have a larger impact on HRQoL [26]. Despite 68% reporting remaining symptoms, only 18% reported a worsening and 95% would accept the surgery if offered it for the same diagnosis.

Due to the scarcity of HRQoL data on spinal schwannomas, other diagnoses were evaluated to provide a basis for comparison. Lumbar spinal stenosis (LSS) and spinal meningiomas share similarities in symptoms, anatomical structures affected, and in the surgical treatments used. Both spinal schwannomas and meningiomas belong to the group of benign intradural extramedullary tumors and are treated using similar approaches [27,28]. The initial symptoms for all three conditions include pain and neurological deficits secondary to compression of the spinal cord or nerves. In fact, Schwannomas may initially be misdiagnosed as LSS [29].

HRQoL was significantly improved following surgery for LSS [27,28]. A study using the Health Utilities Index Mark 3 (HUI3), where 1 represents perfect health, reported that the mean unadjusted overall scores were significantly lower for LSS (0.6) than the general population sample (0.85). Large differences in HRQoL remained after adjustment for sex and age [30]. A study by Pettersson-Segerlind et al. reported that the HRQoL of surgically treated spinal meningioma patients was equal to that of the general population. The EQ5D_index_ and EQ_VAS_ for spinal meningiomas were 0.76 and 74 [31] and 0.8 and 80 for spinal schwannomas (current study), indistinguishable from the general population samples.

In a study on 58 patients with LSS, only 22% returned to work, while 38% did not feel like working again and 28% felt unfit for work. On a 0–100 scale comparable to EQvas, the domain scores for physical health, psychological health, social relations with friends and family or at work ranged from 59 to 61 on the WHOQOL-BREF, considerably lower than the range of 69 to 81 of the general population sample. However, the HRQoL of those who returned to work was similar to that of a healthy normal population [32]. Another study on 439 LSS patients showed that roughly 40% returned to work after surgery [33].

Pettersson-Segerlind et al. also showed that all patients who were working before surgery returned to work, in most cases within 3 months postoperatively [31]. These results are similar to the present study, suggesting that surgery for benign intradural extramedullary tumors is effective and leads to subjective recovery as well as an early return to work.

In summary, this study provides a first report on the HRQoL and return to work in spinal schwannoma patients compared to the general population. The findings provide new insights into the overall health and outcomes after surgery for spinal schwannomas, where previous data are lacking. The long-term, post-surgery, HRQoL of this cohort was comparable to that of the general population and patients with spinal meningiomas and much better than patients with LSS. The patients were satisfied with the surgery and almost all would accept the same treatment if offered it today.

### Strengths and Limitations

The strength of this study is its population-based design and relatively large sample of spinal schwannoma patients. The follow-up duration was sufficient to enable the capture of long-term outcomes [34]. The surgeon bias was limited in this study by institutional routine since all referred cases of spinal schwannomas with imminent or manifest nerve root or spinal cord compression were offered surgery.

The general population sample was retrieved from the same Swedish region and was matched by sex and age since the HRQoL differs between sexes and ages [15]. The general population sample also included respondents with chronic diseases and not only healthy individuals.

Of the 180 consecutive patients, 9 had died and 86 did not participate. While we cannot exclude some selection bias, there were no differences in baseline parameters between those who responded to the questionnaire and those who did not (Supplementary Table S1). The matched controls were selected from a population sample with a 61% response rate, which may also have given an overly favorable assessment of HRQOL in this sample. We consider the data to be representative with external validity for surgically treated spinal schwannoma patients.

Previous studies have shown that non-response bias may affect the results of medical surveys [35,36,37]. The non-response becomes critical when response rates fall below 70%. The likelihood of non-response bias can be assessed by comparing baseline characteristics of responders and non-responders. A similarity in the baseline is reassuring but does not exclude the possibility of bias [38]. Therefore, the results of this study may be interpreted with caution in this regard.

A further limitation was that the study-specific questionnaire was not validated. However, questions included in the questionnaire mainly concerned symptoms, medication, and employment status, which were considered to have face validity.

## 5. Conclusions

Surgery for spinal schwannoma is safe and the results show a significant improvement in neurological function. The HRQoL was equal between the spinal schwannoma sample and the general population. The spinal schwannoma sample reported a limited use of pain medication and patients working preoperatively generally returned to work. Based on the findings of this study, surgical treatment of spinal schwannomas is associated with good HRQoL and with a high frequency of return to work.

## Figures and Tables

**Table 1:** Characteristics of the spinal schwannoma sample.

Variable	Value (*n* = 94)
Male sex	45 (48%)
Age (years) [IQR]	52 [42–64]
Symptom duration (months) [IQR]	12 [6–24]
Charleston comorbidity index	1 [0–2]
Tumor recurrence	6 (6.4%)
Preoperative mMC	
1	35 (37%)
2	53 (56%)
3	6 (6.4%)
4	0 (0.0%)
5	0 (0.0%)
Postoperative mMC	
1	48 (51%)
2	44 (47%)
3	1 (1.1%)
4	0 (0.0%)
5	0 (0.0%)
Missing data	1 (1.1%)
Long-term mMC	
1	61 (65%)
2	32 (34%)
3	1 (1.1%)
4	0 (0.0%)
5	0 (0.0%)

**Table 2:** Percentage (number) of respondents reporting no, moderate and severe problems in EQ-5D dimensions, EQ-5D_index_ and EQ_VAS_, spinal schwannoma sample and general population sample.

	Total					Men					Women				
EQ-5D Dimensions	Schwannoma *n* = 94		General Population *n* = 282		*p* ^a,b^	Schwannoma *n* = 45		General Population *n* = 135		*p* ^a,b^	Schwannoma *n* = 49		General Population *n* = 147		*p* ^a,b^
	%	*n*	%	*n*		%	*n*	%	*n*		%	*n*	%	n	
Mobility					0.469					0.515					0.700
No problems	75.5	71	79.4	224		77.8	35	82.2	111		73.5	36	76.9	113	
Moderate problems	24.5	23	20.6	27		22.2	10	17.8	24		26.5	13	23.1	34	
Severe problems	0.0	0	0.0	0		0.0	0	0.0	0		0.0	0	0.0	0	
Self-care					0.756					1.000					0.713
No problems	95.7	90	96.5	272		97.8	44	97.8	132		93.9	46	95.2	140	
Moderate problems	3.2	3	2.5	7		2.2	1	0.7	1		4.1	2	4.1	6	
Severe problems	1.1	1	1.1	3		0.0	0	1.5	2		2.0	1	0.7	1	
Usual activities					0.092					0.020					1.000
No problems	79.8	75	87.2	246		75.6	34	90.4	122		83.7	41	84.4	124	
Moderate problems	19.1	18	11.3	32		22.2	10	8.9	12		16.3	8	13.6	20	
Severe problems	1.1	1	1.4	4		2.2	1	0.7	1		0.0	0	2.0	3	
Pain/discomfort					0.095					0.302					0.240
No problems	39.4	37	49.6	140		46.7	21	56.3	76		32.7	16	43.5	64	
Moderate problems	55.3	52	45.7	129		48.9	22	40.0	54		61.2	30	51.0	75	
Severe problems	5.3	5	4.6	13		4.4	2	3.7	5		6.1	3	5.4	8	
Anxiety/depression					0.520					0.240					0.863
No problems	67.0	63	70.6	199		66.7	30	76.3	103		67.2	33	65.3	96	
Moderate problems	31.9	30	27.7	78		31.1	14	23.0	31		32.7	16	32.0	47	
Severe problems	1.1	1	1.8	5		2.2	1	0.7	1		0.0	0	2.7	4	
EQ-5D_index_ median (IQR)	0.796 (0.725–1.0)		0.796 (0.727–1.0)		0.095	0.796 (0.725–1.0)		0.848 (0.727–1.0)		0.388	0.796 (0.707–1.0)		0.796 (0.725–1.0)		0.274
EQ_VAS_ median (IQR)	80.0 (68.5–90.0) ^c^		80.0 (70.0–90.0)		0.527	80.0 (63.3–94.3) ^c^		85.0 (70.0–90.0)		0.633	80.0 (70.0–90.0)		80.0 (70.0–90.0)		0.678

^a^ Differences between spinal schwannoma sample and general population sample. ^b^ Moderate and severe levels in EQ-5D dimensions collapsed before Chi-square analysis. ^c^ Missing values, *n* = 1. EQ_index_, EQ_VAS_–md (IQR) = independent samples median test with Yates’s continuity correction. EQ-5D dimensions = Fisher’s exact test.

**Table 3:** Patient reported remaining symptoms and medication.

Variable	Value (*n* = 94)
Remaining symptoms after surgery	
Yes	64 (68%)
No	30 (32%)
Have the symptoms changed after surgery?	
Better	38 (40%)
Unchanged	35 (37%)
Worse	17 (18%)
Missing data	4 (4.3%)
Medications related to surgery	
None	60 (64%)
NSAID or Paracetamol	30 (32%)
Morphine derivates	0 (0.0%)
Neuropathic pain medication	3 (3.2%)
Spasticity medication	0 (0.0%)
Missing data	1 (1.1%)

## Data Availability

Upon reasonable request, the data may be provided by contacting the corresponding author A.E.-T.

## Supplementary Materials

The following supporting information can be downloaded at: https://www.mdpi.com/article/10.3390/cancers16101882/s1, Table S1: Illustrating that there is no bias between responders and non-responders.
