# Supplementary material for: Health-Related Quality of Life and Return to Work after Surgery for Spinal Schwannoma: A Population-Based Cohort Study
Source: Cancers (Basel). 2024 May 15;16(10):1882. doi: 10.3390/cancers16101882 (PMC11120162; doi:10.3390/cancers16101882)
Supplement: Supplementary file 1 [file cancers-16-01882-s001.zip › cancers-2986442-supplementary.pdf]

Supplementary Table S1: Illustrating that there is no bias between responders and non-responders.

Variable	n	Overall n = 180[1]	Non-responders n = 86[1]	Responders n = 94[1]	p-value[2]
**Age (years) (IQR)**	180	53 (42-64)	54 (41-63)	52 (42-63)	0.86
**Sex**	180				0.56
Female		90 (50%)	41 (48%)	49 (52%)	
Male		90 (50%)	45 (52%)	45 (48%)	
**Prior spinal surgery**	180				0.55
No		170 (94%)	83 (97%)	87 (93%)	
Yes		10 (5.6%)	3 (3.5%)	7 (7.4%)	
**Prior spinal radiation**	180				1
No		180 (100%)	86 (100%)	94 (100%)	
Yes		0 (0%)	0 (0%)	0 (0%)	
**Preoperative mMCs**	180				0.47
1		68 (38%)	33 (38%)	35 (37%)	
2		92 (51%)	39 (45%)	53 (56%)	
3		19 (11%)	13 (15%)	6 (6.4%)	
4		1 (0.6%)	1 (0.1%)	0 (0%)	
**Motor deficits**	180				0.77
No		106 (59%)	51 (59%)	55 (59%)	
Yes		74 (41%)	35 (41%)	39 (41%)	
**Sensory deficit**	180				0.88
No		104 (58%)	50 (58%)	54 (57%)	
Yes		76 (42%)	36 (42%)	40 (43%)	
**Postural imbalance**	180				0.85
No		148 (82%)	72 (84%)	76 (81%)	
Yes		32 (18%)	14 (16%)	18 (19%)	
**Incontinence**	180				0.69
No		151 (84%)	73 (84%)	78 (83%)	
Yes		29 (16%)	13 (16%)	16 (17%)	
**Pain**	180				0.13
No		24 (13%)	8 (9.3%)	16 (17%)	
Yes		156 (87%)	78 (91%)	78 (83%)	
**Cervical tumor**	180				0.06
No		151 (84%)	78 (91%)	73 (78%)	
Yes		29 (16%)	8 (9.3%)	21 (22%)	
**Cord-based Location**	180				0.308
Conus		58 (31%)	30 (35%)	28 (30%)	
Spinal cord		130 (69%)	64 (74%)	66 (70%)	
**Dural-based location**	180				0.43

Extradural		12 (6.7%)	8 (9.3%)	4 (4.3%)	
Intradural		148 (82%)	72 (84%)	76 (81%)	
Combined		20 (11%)	6 (7.0%)	14 (15%)	
**Laminectomy – number of levels**	180				0.66
0		3 (1.7%)	1 (0.1%)	2 (2.1%)	
1		30 (17%)	18 (21%)	12 (13%)	
2		96 (53%)	42 (49%)	54 (57%)	
3		44 (24%)	21 (24%)	23 (24%)	
4		4 (2.2%)	3 (3.5%)	1 (1.1%)	
5		2 (1.1%)	1 (0.1%)	1 (1.1%)	
7		1 (0.6%)	0 (0.0%)	1 (1.1%)	
**Laminoplasty**	180				**0.018**
No		97 (54%)	55 (64%)	42 (45%)	
Yes		83 (46%)	31 (36%)	52 (55%)	
**Preserved nerve root function**	180				0.87
0		47 (26%)	21 (24%)	26 (28%)	
1		133 (74%)	65 (76%)	68 (72%)	

[1] **Median (IQR); n (%)**

[2] **Wilcoxon rank sum test; Wilcoxon rank sum exact test; Pearson's Chi-squared test; Fisher's exact test**
